# Glucagon-like Peptide-1 Secretion Is Inhibited by Lysophosphatidic Acid

**DOI:** 10.3390/ijms23084163

**Published:** 2022-04-09

**Authors:** Maria F. Fernandes, Michelle V. Tomczewski, Robin E. Duncan

**Affiliations:** Department of Kinesiology and Health Sciences, Faculty of Health, University of Waterloo, 200 University Ave W., BMH1044, Waterloo, ON N2L 3G1, Canada; maria.fernandes@uwaterloo.ca (M.F.F.); mvtomczewski@uwaterloo.ca (M.V.T.)

**Keywords:** glucagon-like peptide 1, lysophosphatidic acid, L-cells, inflammation

## Abstract

Glucagon-like peptide-1 (GLP-1) potentiates glucose-stimulated insulin secretion (GSIS). While dozens of compounds stimulate GLP-1 secretion, few inhibit. Reduced GLP-1 secretion and impaired GSIS occur in chronic inflammation. Lysophosphatidic acids (LPAs) are bioactive phospholipids elevated in inflammation. The aim of this study was to test whether LPA inhibits GLP-1 secretion in vitro and in vivo. GLUTag L-cells were treated with various LPA species, with or without LPA receptor (LPAR) antagonists, and media GLP-1 levels, cellular cyclic AMP and calcium ion concentrations, and DPP4 activity levels were analyzed. Mice were injected with LPA, with or without LPAR antagonists, and serum GLP-1 and DPP4 activity were measured. GLUTag GLP-1 secretion was decreased ~70–90% by various LPAs. GLUTag expression of *Lpar1*, *2*, and *3* was orders of magnitude higher than *Lpar4, 5*, and *6*, implicating the former group in this effect. In agreement, inhibition of GLP-1 secretion was reversed by the LPAR1/3 antagonist Ki16425, the LPAR1 antagonists AM095 and AM966, or the LPAR2 antagonist LPA2-antagonist 1. We hypothesized involvement of Gα_i_-mediated LPAR activity, and found that intracellular cyclic AMP and calcium ion concentrations were decreased by LPA, but restored by Ki16425. Mouse LPA injection caused an ~50% fall in circulating GLP-1, although only LPAR1 or LPAR1/3 antagonists, but not LPAR2 antagonism, prevented this. GLUTag L-cell and mouse serum DPP4 activity was unchanged by LPA or LPAR antagonists. LPA therefore impairs GLP-1 secretion in vitro and in vivo through Gα_i_-coupled LPAR1/3 signaling, providing a new mechanism linking inflammation with impaired GSIS.

## 1. Introduction

Type 2 diabetes mellitus (T2DM) is considered an inflammatory disease, and there is evidence that inflammation precedes development, suggesting a causal role [1,2]. While mechanisms have been identified that help to explain this association, links between inflammation and impaired glycemic control are not fully understood. Glucagon-like peptide-1 (GLP-1) is a member of the incretin hormones that potentiate glucose-stimulated insulin secretion (GSIS) from beta-cells, allowing for a normal response to glucose ingestion [3]. Chronic inflammation impairs GSIS [4,5,6], and is associated with reduced GLP-1 secretion [7,8,9]. The term lysophosphatidic acid (LPA) encompasses a group of circulating bioactive signaling lipids that are increased in inflammation [10,11], obesity [12], and chronic disease states [13]—conditions where reduced GLP-1 levels and action are also often observed [14]. While numerous classes of nutrients, hormones, neurotransmitters, and other chemicals stimulate GLP-1 secretion, only somatostatin, galanin, and α-adrenergic receptor activation inhibit GLP-1 secretion [3]. We investigated whether lysophosphatidic acid inhibits GLP-1 secretion.

**Figure 1:**
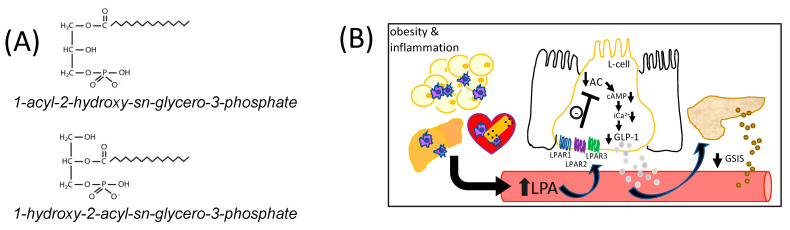
The chemical structure of LPA and model of regulation of GLP-1 secretion. (**A**) The general chemical structure of LPA is shown. LPAs are comprised of a fatty acyl moiety esterified to the sn-1 or sn-2 position of glycerol-3-phosphate. (**B**) This diagram shows the postulated model whereby obesity-derived LPA, and LPA derived from other chronic inflammatory conditions such as non-alcoholic fatty liver disease or cardiovascular disease, inhibits GLP-1 secretion in vivo. LPA was hypothesized to suppress GLP-1 release from intestinal L-cells (shown flanked by absorptive epithelial cells) by activating Gα_i_-mediated coupling primarily with LPAR1, 2, and 3, which would inhibit adenylyl cyclase (AC) activity, reducing both cyclic AMP (cAMP) levels and intracellular calcium (iCa^2+^) mobilization, impairing GLP-1 secretion. Impaired GLP-1 secretion to the blood is expected to inhibit glucose-stimulated insulin secretion (GSIS) from beta-cells of the pancreas.

The term LPA refers to a class of bioactive lysophospholipids with the general formula 1-acyl-2-hydroxy-sn-glycero-3-phosphate (1-acyl-LPA), or 1-hydroxy-2-acyl-sn-glycero-3-phosphate (2-acyl-LPA) (Figure 1A) [11,15]. Plasma LPA concentrations are typically around 1 μM, but can rise to over 10 μM under pathological conditions [10,11]. Although there are multiple pathways for LPA production, Autotaxin, a lysophospholipase D (LPLD) found both in tissues and in circulation [16], is a major regulator of circulating LPA concentrations [13]. Autotaxin is normally responsible for ~40% of total circulating LPA [16]. In inflammatory conditions, however, a rise in LPA occurs when activated platelets secrete phospholipases A1 and A2 (PLA_1_/PLA_2_), generating lysophosphatidylcholine and other lysophospholipids that are substrates for the LPLD-mediated action of circulating autotaxin [17]. Increases in autotaxin linked to disease-related conditions can also contribute to elevated circulating LPA. Adipose-derived autotaxin is upregulated in obesity in both mice and humans [18], while circulating autotaxin levels are a biomarker of fibrosis in non-alcoholic fatty liver disease (NAFLD) [19], and high levels of autotaxin and LPA are found in many different tumor types [20]. This enzyme is also expressed in the necrotic core and fibrous cap of atherosclerotic plaques [21], and evidence suggests these lesions are a direct source of elevated LPA in coronary artery blood [22]. Thus, elevated circulating LPA can be derived from both acute and chronic inflammatory conditions.

Links between glucose intolerance and chronic inflammatory diseases that are sources of LPA, such as obesity [23], NAFLD [24], some cancers [25], and coronary artery disease [26], are well established [27]. In this regard, Rancoule et al. have directly shown that injection of mice with 1-oleoyl-2-hydroxy-sn-glycero-3-phosphate (18:1-LPA) causes impaired glucose tolerance and reduces plasma insulin concentrations [28]. However, a role for LPA in the regulation of incretin hormones, including GLP-1, has not yet been investigated. Indeed, in Rancoule et al.’s study, the reduction in insulin levels following LPA injection was attributed to decreased GSIS from isolated islets, implying a direct effect of LPA on beta-cells [28]. However, indirect actions of LPA and LPAR activation on insulin secretion and glucose control are not precluded by that study. Indeed, in that paper, it was also reported that chronic administration of an LPAR1/3 antagonist increased the number of beta-cells within islets [28]. While this could be a direct effect of modulating LPA-mediated signaling in beta-cells, increases in the number and health of beta-cells could also result indirectly from increased GLP-1 secretion, since GLP-1 improves the health and survival of beta-cells [29,30,31].

GLP-1 is a gastric hormone synthesized and released by enteroendocrine L-cells in the intestinal tract and is one of several proteins that result from alternate post-translational processing of the product of the *Preproglucagon* gene [3,32,33]. It acts through the GLP-1 receptor (GLP-1R) that is found on a variety of cell types [3,34]. In healthy beta-cells, GLP-1 acts in a glucose-dependent manner to potentiate insulin secretion when blood glucose concentrations increase, by activating a rise in cytosolic Ca^2+^ (iCa^2+^) concentrations to promote insulin exocytosis [35,36]. GLP-1 also improves insulin secretory capacity by increasing pro-insulin synthesis [35], promoting maturation of insulin-containing granules to increase the available secretory pool [37], increasing beta-cell proliferation [30,38,39], and protecting against beta-cell apoptosis [29,30,31]. Thus, factors that chronically improve GLP-1 levels may increase the number and function of beta-cells.

L-cell GLP-1 secretion is triggered by a rise in iCa^2+^ concentrations and is stimulated by a variety of dietary factors and hormones [3]. GLP-1 secretion in response to glucose is initially induced by the co-transport of sodium via apical SGLT1 from the lumen, which stimulates membrane depolarization and calcium influx [40]. This is augmented by the rise in ATP generated from glucose metabolism that is supported by GLUT2-mediated glucose uptake [41]. Thus, both oral glucose and glucose entering L-cells from the basal side following intraperitoneal (*i.p.*) injections can stimulate GLP-1 secretion [3]. The rise in ATP closes K_ATP_ channels, which triggers membrane depolarization and opening of voltage-dependent Ca^2+^ channels, resulting in a rapid influx of calcium that triggers exocytosis and secretion of GLP-1 [42]. Fatty acids and 2-monoacylglycerol generated from triacylglycerol hydrolysis can also stimulate Ca^2+^-dependent GLP-1 secretion via binding to GPR120, GPR40, and GPR119 [43,44], while proteins and certain amino acids can activate GLP-1 secretion through activation of phosphoinositide-3-kinase, MEK1/2, and p38 kinase-dependent pathways, that also lead to a rise in iCa^2+^ [3]. GLP-1 secretion is also stimulated by cholinergic neuroendocrine-mediated signaling [45], by beta-adrenergic receptor-mediated signaling that raises cellular cAMP levels [46], by interleukin-6 that is generated during exercise [47], and by the hormones ghrelin [48] and gastrin-releasing peptide [45]. In contrast, only somatostatin, galanin, and α-adrenergic receptor activation have thus far been identified as negative regulators of GLP-1 secretion [3].

We hypothesized that LPA would inhibit GLP-1 release from L-cells through Gα_i_-mediated coupling primarily with LPAR1, 2, and 3, which would inhibit adenylyl cyclase activity to reduce cAMP levels and iCa^2+^ mobilization needed for secretion, and this mechanism is illustrated in Figure 1B. Results from this work provide a new mechanism with broad implications for understanding associations between inflammation and dysglycemia.

## 2. Results and Discussion

### 2.1. LPA Inhibits GLUTag L-Cell GLP-1 Secretion via LPAR1/2/3

**Figure 2:**
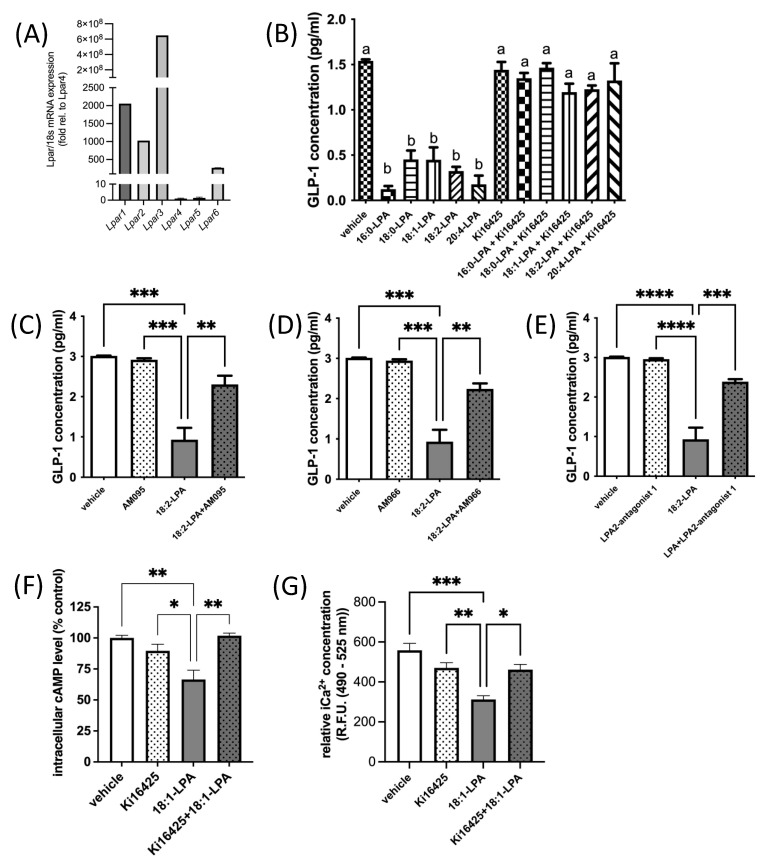
LPA inhibits GLP-1 secretion from GLUTag L-cells. (**A**) Relative mRNA expression of *Lpar1*, *Lpar2, Lpar3, Lpar4, Lpar5, Lpar6* in GLUTag cells. Data are shown relative to the least abundant transcript, *Lpar4* (*n* = 10). (**B**) Treatment of GLUTag cells for 2.5 h with various 1-acyl-LPA species (2.5 µM) significantly inhibits GLP-1 secretion, but this inhibition is prevented when the LPAR1/3 antagonist Ki16425 (10 µM) is added in the last 2 h of incubation (*n* = 3). (**C**–**E**) Three additional LPAR antagonists were found to significantly restore GLP-1 secretion in GLUTag cells treated with 1-linoleoyl-LPA (18:2-LPA; 2.5 µM) for 2.5 h, either alone or with the addition of 10 µM LPAR antagonist (as indicated) after the first 30 min (*n* = 3). (**F**) Intracellular cAMP levels were significantly decreased in GLUTag cells by 2 h treatment with 1-oleoyl-LPA (18:1-LPA; 2.5 µM), and significantly restored by 10 µM Ki16425 co-treatment, although this inhibitor did not significantly affect cAMP levels alone (*n* = 4). (**G**) LPA significantly decreased relative iCa^2+^ levels in GLUTag cells after 2.5 h treatment, relative to cells treated with vehicle or 10 µM Ki16425 alone, which did not significantly affect iCa^2+^ levels. However, adding Ki16425 to 18:1-LPA-treated cells significantly restored iCa^2+^ levels (*n* = 4). Data are means ± SEM. ^a,b^ Bars with different superscripts are significantly different, ^a,b^ *p* < 0.05. * *p* < 0.05, ** *p* < 0.01, *** *p* < 0.001, and **** *p* < 0.0001. Differences were analyzed by 1-way ANOVA with Bonferroni’s post hoc test.

Six known LPARs have been characterized [49,50]. LPAR1–3 are members of the endothelial differentiation gene (Edg) family of GPCRs that share ~50% amino acid sequence homology [11,15,51,52], while LPAR4–6 are part of the purinergic-like family, sharing ~35% homology [11,53,54]. LPAR1, 2, 3, 4, and 6 are all Gα_i_-coupled [49,50]. We found that all six canonical *Lpar* are expressed in murine GLUTag L-cells (Figure 2A). However, *Lpar1*, *2*, and *3* are expressed at levels that are orders of magnitude higher than *Lpar4*, *5*, or *6* (Figure 2A). In agreement with our hypothesis, treatment of GLUTag L-cells with a variety of different 1-acyl-LPA species (2.5 μM) for 2.5 h caused GLP-1 levels in the surrounding media to decrease by two-thirds, or more (Figure 2B). The level of inhibition did not appear to be related to chain length or degree of saturation of fatty acyl species in the LPAs tested, although future studies could examine additional species for relative activity. Addition of the LPAR1/3 antagonist Ki16425 (10 μM) 30 min after addition of LPA completely prevented this decrease in GLP-1 (Figure 2B). Ki16425 acts predominantly on LPAR1 and LPAR3, with respective K_i_ values of 0.34 μM and 0.93 μM, while the K_i_ for LPAR2 is 6.5 μM. Notably, Ki16425 treatment alone did not significantly alter GLP-1 secretion, indicating a specific restoration of the inhibitory effect of LPA, rather than a non-specific stimulatory effect.

The involvement of LPAR was further investigated by testing the efficacy of additional antagonists (Figure 2C–E). In L-cells treated with 2.5 μM 18:2-LPA, a significant decrease in media GLP-1 concentration was observed, as expected. Similar to effects observed with Ki16425, treatment of cells with 10 μM AM095, AM966, or LPA2-antagonist 1 caused a significant restoration of GLP-1 concentrations by GLUTag cells. AM095 is highly selective for LPAR1, with an IC50 concentration for inhibition of less than 1 μM [55]. In an intracellular calcium release assay, AM966 has been found to have IC50 values of 17 nM, 1.7 μM, 1.6 μM, 7.7 μM, and 8.6 μM for LPAR1–5, respectively [56]. LPA2-antagonist 1 acts predominantly through LPAR2 with an IC50 value of only 17 nM, compared with IC50 values > 50 μM for LPAR1 and LPAR3 [57]. Similar effects among the three inhibitors suggest that antagonism of LPAR1, 2, and 3 may all help to relieve LPA-mediated inhibition of GLP-1 secretion. However, given the greater abundance of *Lpar1*, which was ~2-fold more abundant than *Lpar2*, and *Lpar3*, which was ~10^5^-fold more abundant than *Lpar2* (Figure 2A), it is possible the predominance of the receptor species present may affect the net outcome of signaling.

We next examined whether the hypothesized LPA-mediated decrease in cAMP concentrations in GLUTag cells would be evident, and whether this effect would be restored by LPAR1/3 receptor antagonism. GLUTag cells treated with 18:1-LPA for 2 h had significantly lower cAMP concentrations than control cells treated with vehicle alone, but this effect was reversed to control cell levels when cells were treated with 18:1-LPA in the presence of Ki16425 (Figure 2F). This effect corresponded with changes in calcium mobilization, wherein cells treated with 18:1-LPA for 2.5 h had significantly lower iCa^2+^ concentrations, but this effect of LPA was reversed by the addition of Ki16425 for the last 2 h of the incubation (Figure 2G).

### 2.2. LPAR1/3 Antagonists Reverse LPA-Mediated Decreases in Mouse Serum GLP-1 Levels 

**Figure 3:**
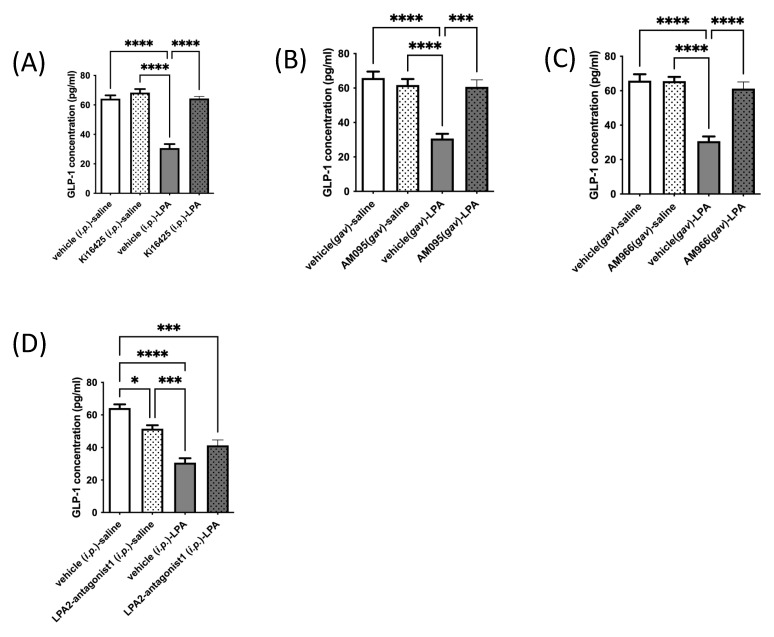
LPA-mediated decreases in circulating GLP-1 levels in mice are significantly restored by LPAR1/3 inhibitors. Circulating GLP-1 levels were analyzed in mice. Animals were injected *i.p.* with vehicle control (10% DMSO), Ki16425 (5 mg/kg), or LPA2-antagonist 1 (5 mg/kg), or gavaged with vehicle control (10% DMSO), AM095 (30 mg/kg), or AM966 (30 mg/kg), followed by *i.p.* injection with vehicle control (isotonic saline) or 18:1-LPA (50 mg/kg) 10 min later. Blood was taken thirty minutes following the last injection. Reduced circulating levels of GLP-1 in mice treated with LPA were restored to control values upon treatment with the LPAR1/3 antagonist Ki16425 (5 mg/kg) (**A**), the LPAR1 antagonist AM095 (30 mg/kg) (**B**), or the LPAR1 antagonist AM966 (30 mg/kg) (**C**). Reduced blood GLP-1 levels were not significantly rescued by LPA2-antagonist 1 (**D**). Data are means ± SEM (*n* = 5). Statistically significant differences were analyzed by 1-way ANOVA with Bonferroni’s post hoc test, * *p* < 0.05, *** *p* < 0.001, and **** *p* < 0.0001.

Based on effects observed in GLUTag L-cells in culture, we next tested whether an in vivo response occurs by injecting or gavaging mice with various LPAR antagonists or vehicle control (administered through the same route as the drug), followed 10 min later by an injection of 18:1-LPA, or vehicle (isotonic saline). Similar to effects observed in cultured L-cells, treatment of mice with LPA caused a significant and dramatic fall in circulating GLP-1 concentrations, by approximately half (Figure 3A–D). Additionally, similar to effects seen in culture, Ki16425, AM095, and AM966 each had no significant effect on serum GLP-1 concentrations relative to vehicle control, but fully prevented the LPA-mediated decline (Figure 3A–C). Interestingly, however, a different effect was observed in vivo for LPA2-antagonist 1 than in vitro (Figure 2E and Figure 3D, respectively). This LPAR2-specific antagonist significantly decreased circulating GLP-1 concentrations by approximately 20% when given alone, and did not significantly raise GLP-1 concentrations in mice treated with LPA (Figure 3D). The reason for the discrepancy between in vitro and in vivo effects is not immediately apparent, but suggests that the LPAR expression profile of L-cells in mice may differ from that of GLUTag cells. It is also possible that the difference relates to the complexity of action of LPAR2 antagonism in vivo, where activity on multiple cell and organ types could result in effects that alter circulating GLP-1, independent of effects on L-cell secretion.

### 2.3. DPP4 Activity in GLUTag L-Cells and Mouse Serum Is Not Altered by LPA or LPAR1/3 Antagonism 

**Figure 4:**
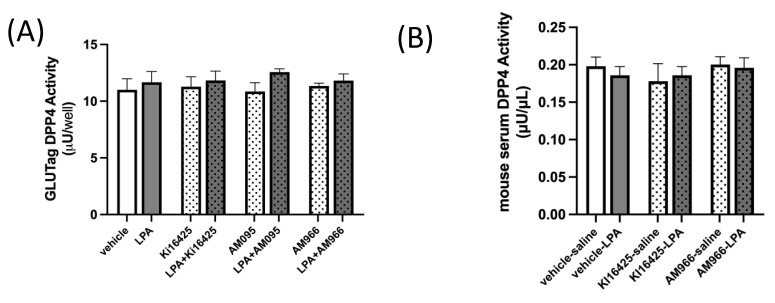
DPP4 activity is not affected by LPA or LPAR antagonists in GLUTag cells or mice. (**A**) DPP4 activity is not significantly affected by treatment of GLUTag cells with 18:1-LPA (2.5 µM) for 2.5 h, whether given alone or in combination with 10 µM levels of the LPAR antagonists Ki16425, AM095, or AM966, which also did not affect DPP4 activity (*n* = 4). (**B**) DPP4 activity in serum is not significantly affected either by treatment of mice with 18:1-LPA, alone or in combination with the LPAR antagonists Ki16425 (5 mg/kg) or AM966 (30 mg/kg) (*n* = 5). Data are means ± SEM.

It is important to consider whether LPA may affect GLP-1 clearance. Dipeptidylpeptidase-4 (DPP4) can cleave active GLP-1 to generate inactive forms of GLP-1, which have a low affinity for the GLP-1 receptor (GLP-1R) and are efficiently cleared by the kidneys [3]. DPP4 is found both on the surface of cells and in the circulation, and deactivating cleavage of GLP-1 by DPP4 is a major regulator of activity [3]. We assayed DPP4 activity in GLUTag cells treated with LPA, with and without various LPAR1 or LPAR1/3 inhibitors (Figure 4A), and also in the same mouse serum used to assess GLP-1 levels in Figure 2 (Figure 4B), but found no significant effects of either LPA treatment or LPAR antagonism, indicating that changes in DPP4-mediated GLP-1 degradation are unlikely to be an important factor in mediating the changes in GLP-1 concentrations observed.

### 2.4. Summary and Future Directions

To the best of our knowledge, this is the first study to demonstrate that LPA inhibits GLP-1 secretion through LPAR1–3 agonism. Our findings therefore indicate that GLP-1 secretion is inhibited by an inflammatory class of lipid species, and provide a new mechanism to potentially explain impaired GSIS in several chronic disease conditions [18,19,20,21]. Most obviously, an impaired incretin effect is known to occur early in T2DM [14], and impaired GLP-1 secretion is considered a mechanism in this effect [58]. Indeed, GLP-1R agonists and DPP4 inhibitors are important therapeutics in T2DM management. The response of beta-cells to a rise in blood glucose is modulated by the action of gut-derived incretin hormones, including GLP-1, that potentiate GSIS [59]. However, chronic hyperglycemia in T2DM can cause beta-cell incretin resistance [60]. Beta-cell GLP-1 resistance raises the level of GLP-1 that is needed for effective beta-cell signaling, but deteriorating glycemic control is associated with reduced GLP-1 secretion in this state [61]. When there is insufficient GLP-1 to potentiate GSIS, hyperglycemia is worsened, fueling a vicious cycle. Our findings suggest that LPAR antagonism could be a novel therapy to help break this cycle, by relieving an inflammation-associated block on GLP-1 secretion.

GLP-1 has health benefits beyond improving GSIS and reducing hyperglycemia. GLP-1Rs are present in the heart and vascular endothelium, and GLP-1R agonists can improve cardiac contractility and output and protect against myocardial ischemic injury [62,63,64,65,66]. A consensus statement from the European Society of Hypertension’s working group on ‘Obesity, Diabetes, and the High-Risk Patient’ indicates that GLP-1R agonists can also offer renal and liver protection beyond effects on blood glucose lowering [67]. There is also evidence that GLP-1R agonists may offer some neuroprotection against the development of Alzheimer’s disease and other dementias [68,69,70,71]. Strategies to prevent declines in GLP-1, and/or raise endogenous GLP-1 levels to an effective range, are thus likely to have clinical benefits beyond improving blood glucose regulation. Our findings therefore suggest that raising GLP-1 through LPAR1–3 antagonism could have novel therapeutic implications for a variety of diseases influenced by this hormone.

## 3. Materials and Methods

### 3.1. Materials

Lysophosphatidic acid species used were 1-palmitoyl-2-hydroxy-sn-glycero-3-phosphate (sodium salt) (16:0-LPA; Cat. No. 857123), 1-stearoyl-2-hydroxy-sn-glycero-3-phosphate (sodium salt) (18:0-LPA; Cat. No. 857128), 1-linoleoyl-2-hydroxy-sn-glycero-3-phosphate (sodium salt) (18:2-LPA; Cat. No. 857138), and 1-arachidonoyl-2-hydroxy-sn-glycero-3-phosphate (ammonium salt) (20:4-LPA; Cat. No. 857125-C) from Avanti Polar Lipids (Millipore Sigma, Mississauga, ON, Canada), and 1-oleoyl-2-hydroxy-sn-glycero-3-phosphate (18:1-LPA; Cat. 10010093) from Cayman Chemical (Ann Arbor, MI, USA). LPAR antagonists utilized were Ki16425 (Cat. No. A10501, AdooQ BioSciences, Irvine, CA, USA.), AM966 (Cat. No. A13982, AdooQ BioSciences), AM095 (Cat. No. 22141, Cayman Chemical), LPA2-antagonist 1 (Cat. No. 22051, Cayman Chemical).

### 3.2. Cells

GLUTag cells were a kind gift from Dr. D. J. Drucker (University of Toronto, Toronto, ON, Canada), and were routinely sub-cultured in low-glucose Dulbecco’s modified Eagle’s medium (DMEM, Cat. No. D6046; Millipore Sigma, Mississauga, ON, Canada) supplemented with 10% fetal bovine serum (*v*/*v*) (FBS, Cat. No. A4766801; Millipore Sigma) and 1% (*v*/*v*) penicillin–streptomycin (PS, Cat. No. SV30010; Thermo Fisher Scientific, Mississauga, ON, Canada). Cells were incubated at 37 °C in 5% CO_2_ until 70–80% confluent. Different passages of cells were used to generate biological replicates for measurements. When replicate measures from the same passage were performed, these were considered technical replicates, and were averaged to generate a single mean for that biological replicate.

### 3.3. RT-qPCR and Gene Expression Analysis

Total RNA was extracted using Trizol^®^ from GLUTag cells grown to 70–80% confluency and reverse-transcribed as previously described using SuperScript II Reverse Transcriptase (Invitrogen, Burlington, ON, Canada) [72]. Gene expression was analyzed using Taqman™ gene expression assays with primer amplification efficiency calculated at 100 ± 2%, allowing for relative comparisons, using the following primers sets: *Lpar1*: Mm01346925_m1, *Lpar2*: Mm00469562_m1, *Lpar3*: Mm00469694_m1, *Lpar4*: Mm02620784_s1, *Lpar5*: Mm02621109_s1, *Lpar6*: Mm00613058_s1; *18S*: Mm03928990_g1 (Applied Biosystems, Mississauga, ON, Canada) using PerfeCTA qPCR FastMix (QuantaBiosciences, Gaithersburg, MD, USA; Cat. No. 95076). Gene expression was analyzed using the ΔΔCt method, with expression normalized to *18 s* as a loading control, and then calculated relative to the least abundant transcript, *Lpar4.*

### 3.4. GLUTag L-Cell GLP-1 Secretion Assay

GLUTag cells (0.3 × 10^6^ cells) were cultured in 6-well plates until ~80% confluent. The growth medium was replaced with low-glucose DMEM containing charcoal-stripped FBS (Millipore Sigma, Mississauga, ON, Canada), and cells were treated with either vehicle (0.05% DMSO (*v*/*v*)), or various LPA species (2.5 μM), and growth was continued for 30 min, after which LPAR antagonists were added (10 μM final concentration) for an additional 2 h. Samples of media were then assayed to determine GLP-1 concentrations using a GLP-1 enzyme immunoassay kit (RAB0201; Millipore Sigma, Mississauga, ON, Canada) according to the manufacturer’s protocol.

### 3.5. Intracellular Ca^2+^ Assay

GLUTag cells were seeded into a 96-well plate and grown to 50–70% confluence. Intracellular calcium was measured using Fluo 4 acetoxymethyl ester (Fluo4 AM; Thermo Fisher Scientific, Mississauga, ON, Canada), which was reconstituted in anhydrous DMSO at a stock concentration of 5 mM, then diluted to 1 μM in media containing charcoal-stripped FBS (10%) immediately prior to the experiment. Cells were incubated in 100 μL Fluo4 AM (1 μM final concentration) for 10 min, then the Fluo4 AM-containing media were removed and replaced with media containing charcoal-stripped FBS (10%) and lacking the fluorophore. Fluorescence was read at 490–525 nm using a BioTek Synergy™ H1 microplate reader (Agilent Technologies Canada, Mississauga, ON, Canada).

### 3.6. Intracellular cAMP Levels

The intracellular cAMP levels were measured using a cAMP-Glo™ Max Assay (Promega, Madison, WI, USA) kit according to the manufacturer’s protocol. GLUTag cells were seeded in a white 96-well plate at an initial density of 10,000 cells per well. The following day, the media were removed and replaced with the complete induction buffer (low-glucose DMEM containing 100 μM Ro 20–1724 and 500 μM IBMX) and vehicle control (0.05% DMSO (*v*/*v*)) or 2.5 μM 18:1-LPA with or without 10 μM Ki16425 for 2 h at 37 °C, 5% CO_2_. Cells were then lysed with the cAMP Detection Solution for 20 min at room temperature. Finally, 50 μL Kinase-Glo^®^ Reagent was added to each well and incubated for 10 min at room temperature before measuring luminescence using a BioTek Synergy™ H1 microplate reader (Agilent Technologies). The cAMP level was normalized to vehicle-treated control cells.

### 3.7. Mouse Serum GLP-1 Assay

Experiments were performed in 16–18-week-old C57Bl/6J male mice, housed in a temperature- and humidity-controlled environment on a 12:12 h light/dark cycle. Mice were bred at the University of Waterloo Central Animal Facility from founders obtained from Jackson Laboratories (Bar Harbor, ME, USA). All animal procedures were approved by the University of Waterloo Research Ethics Board—Animal Care Committee (AUPP#42946 (approval date 9 June 2021), AUPP#18-03 (27 February 2018)) and complied with guidelines of the Canadian Council on Animal Care. At timepoint 0, mice were injected *i.p.* with vehicle control (10% DMSO), Ki16425 (5 mg/kg), or LPA2-antagonist 1 (5 mg/kg), or were gavaged with vehicle control (10% DMSO), AM095 (30 mg/kg), or AM966 (30 mg/kg). Ten minutes later, mice were injected *i.p.* with vehicle control (isotonic saline) or 18:1-LPA (50 mg/kg). Thirty minutes later, mice were euthanized by cervical dislocation and whole blood was rapidly collected by cardiac puncture. Blood was allowed to clot and centrifuged at 1000× *g* for 10 min in a refrigerated centrifuge. Serum was collected and stored at −20 °C until assay for GLP-1 secretion using a GLP-1 EIA Kit (RAB0201; Millipore Sigma, Mississauga, Ontario, Canada). Optimal doses of LPA and LPAR inhibitors were determined from pilot studies.

### 3.8. GLUTag L-Cell DPP4 Activity Assay

DPP4 activity was measured using a DPP4 fluorometric activity assay kit (Abcam kit# ab204722, Toronto, ON, Canada). GLUTag cells were grown to a density of 2 × 10^6^ cells per well according to the manufacturer’s protocol, and then treated in media containing charcoal-stripped FBS (10%) with either no treatment control, vehicle (0.05% DMSO), or 2.5 μM 18:1-LPA for 30 min, after which the LPAR inhibitors were added as indicated (10 μM final concentration), for an additional 2 h. Cells were then harvested and assayed according to the manufacturer’s protocol.

### 3.9. Mouse Serum DPP4 Activity Assay

Mice were administered LPAR antagonists or vehicle control followed by LPA or saline and then euthanized for blood collection, as described in the method for measurements of GLP-1 release in mice. Blood was stored at −80 °C until it was assayed. DPP4 enzymatic activity was measured using a DPP4 fluorometric activity assay kit (Abcam kit# ab204722, Toronto, ON, Canada) according to the manufacturer’s instructions.

### 3.10. Statistical Analysis

Data shown are means ± S.E.M. Differences between groups in levels of GLP-1, cAMP levels, iCa^2+^ levels, and DPP4 activity levels were analyzed by 1-way ANOVA with Bonferroni’s post hoc test.

## Data Availability

Data are available upon reasonable request to the authors.
